# Supplementary material for: NuRD subunit CHD4 regulates super-enhancer accessibility in rhabdomyosarcoma and represents a general tumor dependency
Source: eLife. 2020 Aug 3;9:e54993. doi: 10.7554/eLife.54993 (PMC7438112; doi:10.7554/eLife.54993)
Supplement: Supplementary file 1. [file elife-54993-supp1.docx]

**Sequence of guide RNAs used for the NuRD-centered CRISPR screen.**

| Target | Guide Number | Sequence |
| --- | --- | --- |
| CHD3 | 1 | ACGAGGGAGTACTCGGGCGCGGG |
|  | 2 | GGAGTACTGCCGCGTATGCAAGG |
|  | 3 | CTCGCCGGAGCCATAGTCCAGGG |
|  | 4 | GAATTCATTCTCACGAATGATGG |
|  | 5 | GCGCATCGATGGTGGTATCACGG |
| CHD4 | 1 | CTCCTCACTGCCCGCCGAGCAGG |
|  | 2 | TTAGGGATTTTAGGGTCCCGAGG |
|  | 3 | AAGAGGGGCGCTCACTAGGAAGG |
|  | 4 | ATGTATGTCGTAACCTATGTGGG |
|  | 5 | TATAAATACGAACGCATCGATGG |
| MBD2 | 1 | ATGCGCGCGCACCCGGGGGGAGG |
|  | 2 | CGGCGACTCCGCCATAGAGCAGG |
|  | 3 | CGCCACTCGGGGGACGGCCGCGG |
|  | 4 | CCTTCTTCCATCCGGGGGGGAGG |
|  | 5 | GCCTCAGTTGGCAAGGTACCTGG |
| MBD3 | 1 | GAGTGCCCGGCGCTCCCGCAGGG |
|  | 2 | CTTGCCCGTGCGGAAGTCGAAGG |
|  | 3 | GCTTGAAGATGGACGCCGTCTGG |
|  | 4 | CAGCAATGTCGAAGGCGTTCAGG |
|  | 5 | CGTGGTGTTGAGCCATACGCCGG |
| HDAC1 | 1 | CGCAGACGCAGGGCACCCGGAGG |
|  | 2 | TGAGTCATGCGGATTCGGTGAGG |
|  | 3 | TTACGTCAATGATATCGTCTTGG |
|  | 4 | TATTCACCATGGTGACGGCGTGG |
|  | 5 | CATCAATCCCGTCTCGGAGCGGG |
| HDAC2 | 1 | TGGGTCATGCGGATTCTATGAGG |
|  | 2 | TACAACAGATCGTGTAATGACGG |
|  | 3 | GATGTATCAACCTAGTGCTGTGG |
|  | 4 | CTCATTATCTGGTGATAGACTGG |
|  | 5 | TCCGTAATGTTGCTCGATGTTGG |
| MTA1 | 1 | CGATTCTCCGGATCAGGTATGGG |
|  | 2 | CTGCTATAAGGCCGGACCGGGGG |
|  | 3 | CGACTCGGTCTCGTTGAGCAGGG |
|  | 4 | TCGAGTAGGAAACCGGTACCAGG |
|  | 5 | ATCAGAGGCCAACCTTTTCGAGG |
| MTA2 | 1 | TTACCTGGTTAGACGGATTGAGG |
|  | 2 | TGTCTGTCTTTTCCGGCGCAGGG |
|  | 3 | GATCCCAGATCGCCTAGTAGAGG |
|  | 4 | GCAAAGGAACGGCTACGACCTGG |
|  | 5 | GGACTTCAATGATATTCGCCAGG |
| MTA3 | 1 | GCAACCCATACCTAATAAGAAGG |
|  | 2 | TGCGAGCATTATAAGTGTGTTGG |
|  | 3 | CTCTGCCCGCAACACATATCAGG |
|  | 4 | TCAATCTGTCGATCCGTAAGTGG |
|  | 5 | AGTGTCTTAGTACCACTCGGAGG |
| RBBP4 | 1 | TTCTTCCACTGCGTCGTCGAAGG |
|  | 2 | GTCTTTGTTGCGATGATACAAGG |
|  | 3 | AGACTCATGGAGTAGATGCCAGG |
|  | 4 | CAGTGTGAGCATCAACTGAGTGG |
|  | 5 | CAGTCTGCGATCAGTACCACTGG |
| RBBP7 | 1 | TACACCGTTTCTATATGACCTGG |
|  | 2 | GCCCTTCATTGGCTAGTGCTGGG |
|  | 3 | GGTGGCTTTGGTTCTGTAACAGG |
|  | 4 | TGTCTGTGGGATATAAACGCAGG |
|  | 5 | CTGCTGCACGAGTCATTGTTTGG |
| GATAD2A | 1 | CCGAAGAAGCATGCCGAACACGG |
|  | 2 | GCGGCAGAGTCAAATACAAAAGG |
|  | 3 | GCGGCCGCCTGTCGGCTTGCTGG |
|  | 4 | AACTCGTTGTTGGCGGCGCTGGG |
|  | 5 | AGACGGACTTCACGTGCCGCTGG |
| GATAD2B | 1 | GAATCTGTTGAAGCGGAGCTTGG |
|  | 2 | ACGATTGGAAGAAGCCCGACTGG |
|  | 3 | GGCAGCATCAGTCATGGCGCTGG |
|  | 4 | CATGTAGATGAACTCGCTATTGG |
|  | 5 | GGTGAAATCTGTGCGGCACTGGG |
| LSD1 | 1 | CGGACCCCGGCGGTTCCGCCAGG |
|  | 2 | GTCGGACCAGCCGGCGCAAGCGG |
|  | 3 | TGAGAAGTCATCCGGTCATGAGG |
|  | 4 | CGAGTTGCCACATTTCGCAAAGG |
|  | 5 | GAACTCGGCAGTAATATCTCTGG |
| AAVS1 |  | GGGGCCACTAGGACAGGAT |

**Donor DNA sequences used in the CRISPR/Cas9-mediated Flag knockins.**

| Name | Sequence |
| --- | --- |
| N-terminus  3xFlag-CHD4 | GGGTGGGGGCAGGCTGTTGTTCAAAAGTGCATCCATATCCTCCTCCTCAC  TGCCCGCCGAGCAGGGGGACGGGGAGCCCAGGCCCGACGCTTTATCGTCA  TCATCTTTGTAGTCCTTGTCATCATCGTCCTTATAGTCCTTATCGTCGTC  ATCCTTGTAATCCATCCCCTTCCGCTCCCGGCCAGGGAATTGGCCCAGCT |
| C-terminus  3xFlag-CHD4 | TATCAGTGAATTCCCTTGAGATTGGGTGGATTCCTATCATCTGGAATTCT  GACTCCTCGGGTCTCCTTCTCTAGGTAGCACAGCAACAGGATTACAAGGA  TGACGACGATAAGGACTATAAGGACGATGATGACAAGGACTACAAAGATG  ATGACGATAAATAGAGCAGTGAAGATGCAGACTGATACCACCTCCACCGC |
| N-terminus  3xFlag-BRD4 | TTGGGCCTGGGCCTGTGTTGTAGACATTTGGGAAGTTTCTAGTCCATCCC  CCATTACTGGCAGATTTCTCAATCTCGTCCCGGGGCCGCTCTCCGCAGAC  TTGTCGTCATCGTCTTTGTAGTCCTTGTCGTCATCGTCTTTGTAGTCCTT  GTCGTCATCGTCTTTGTAGTCCATGCTAGTGATCCCATCACATTCTTCAC |
